# TIM-3 in Cardiovascular Disease

**DOI:** 10.1007/s11897-026-00755-y

**Published:** 2026-04-07

**Authors:** Laura I. Yousif, Aukje G. Sijtema, Margot J. Chevalier, Rudolf A. de Boer, Wouter C. Meijers

**Affiliations:** Erasmus MC, Cardiovascular Institute, Thorax Center, Department of Cardiology, Rotterdam, The Netherlands

**Keywords:** TIM-3, Immune Checkpoints, Cardio-Immunology, Cardiovascular Disease, Myocardial Infarction, HMGB1

## Abstract

**Purpose of review:**

We summarise the current knowledge of T-cell immunoglobulin and mucin domain-containing protein 3 (TIM-3) across innate and adaptive immune cells and compile emerging evidence in cardiovascular disease (CVD).

**Recent Findings:**

Immune checkpoints have come to light as potent regulators of immune responses in tumour biology, autoimmune disease and, more recently, in CVD. TIM-3 is a complex immune checkpoint expressed on both immune and non-immune cells. It has four known ligands, two of which are only available for binding upon cell damage or death, and binding can be either stimulatory or inhibitory. It functions as a context-dependent modulator of immune reactions in atherosclerosis, myocardial infarction and myocarditis.

**Summary:**

TIM-3 may exert functions in the cardiovascular system, but more mechanistic research is required to investigate whether interference with TIM-3 signalling can be used to improve cardiovascular health.

## Introduction

The immune system is intrinsically linked to cardiovascular health. It serves to support cardiac homeostasis by, among other things, clearing dying cells, eliminating pathogens, and promoting tissue repair [1]. However, severe or prolonged immune activation can cause irreversible damage and drive pathological processes underlying cardiovascular diseases (CVD). Consistent with this dual role, several immune pathways have therefore been directly implicated in cardiovascular pathology, including inflammasome activation, ferroptosis-mediated cell death, and, more recently, immune checkpoint signalling [2, 3]. Immune checkpoints are inhibitory receptors that have predominantly been described to maintain self-tolerance while regulating the duration and intensity of immune-cell activation [4, 5]. The most well-studied immune checkpoints are Cytotoxic T-Lymphocyte-Associated Protein 4 (CTLA-4) and Programmed Cell Death Protein-1 (PD-1), due to their role in tumour immune evasion and the development of immune checkpoint inhibitors (ICI) to boost an immune-mediated response to tumours [4, 6–9]. Immune checkpoint signalling was implicated in the cardiovascular system due to fatal cardiovascular immune-related adverse events in patients treated with ICI [10–14]. Mechanistic studies have implicated specific immune cells and autoimmune-like responses as drivers of ICI-mediated cardiotoxicity, but much remains to be elucidated (e.g. direct cardiac cell interaction or biomarkers for risk prediction) [15–19]. One risk factor for ICI-mediated myocarditis is combination therapy of ICI, which is prescribed to raise efficacy against tumours expressing more than one immune checkpoint ligand. Additionally, next-generation ICI are being developed to treat tumours, including those targeting T-cell immunoglobulin and mucin domain-containing protein 3 (TIM-3). TIM-3 is broadly expressed across both innate and adaptive immune cells, as well as on tumour cells. Within the tumour microenvironment (TME), TIM-3 is frequently upregulated in various cancer types, contributing to T-cell exhaustion, thereby facilitating tumour progression [20]. Inhibition of TIM-3 has therefore been shown to restore anti-tumour T-cell activity, leading to the development of several anti-TIM-3 monoclonal antibodies currently under investigation as potential ICI therapies [21]. Although TIM-3 is primarily considered an inhibitory receptor, several studies have also suggested stimulatory effects in multiple cell types, making its overall role more complex and not yet fully understood [22].

Importantly, a growing body of evidence links TIM-3 to cardiovascular pathologies, with its upregulation observed across multiple CVDs [23–27]. In this review, we provide insights on TIM-3 within the complex immune system and compile the emerging evidence of TIM-3 in CVD.

## TIM-3 Protein

TIM-3 is a member of the TIM family, which plays key roles in immune regulation, including responses in allergy, asthma, transplant tolerance, autoimmunity, and viral infections [28]. Encoded on chromosome 5q33.2 by the *HAVCR2* gene, TIM-3 is a type I transmembrane glycoprotein, structurally characterised by an extracellular immunoglobulin variable (IgV)–like domain, a mucin domain, a single transmembrane helix, and a cytoplasmic tail. The IgV domain, located distal to the membrane, features a deep ligand-binding pocket formed by the FG–CC’ loops, stabilised by conserved cysteine residues, which is essential for specific ligand interactions [28, 29]. The membrane-proximal mucin domain is heavily glycosylated with O- and N-linked glycans, while the transmembrane segment anchors the receptor in the cell membrane. Notably, TIM-3 does not have the classical inhibitory signalling motifs or structural features required to recruit inhibitory phosphatases within its cytoplasmic tail [30, 31]. Instead, it contains six tyrosine residues, of which Y256 and Y263 are phosphorylated upon ligand binding to initiate phosphotyrosine-dependent signalling [29].

**Fig. 1:**
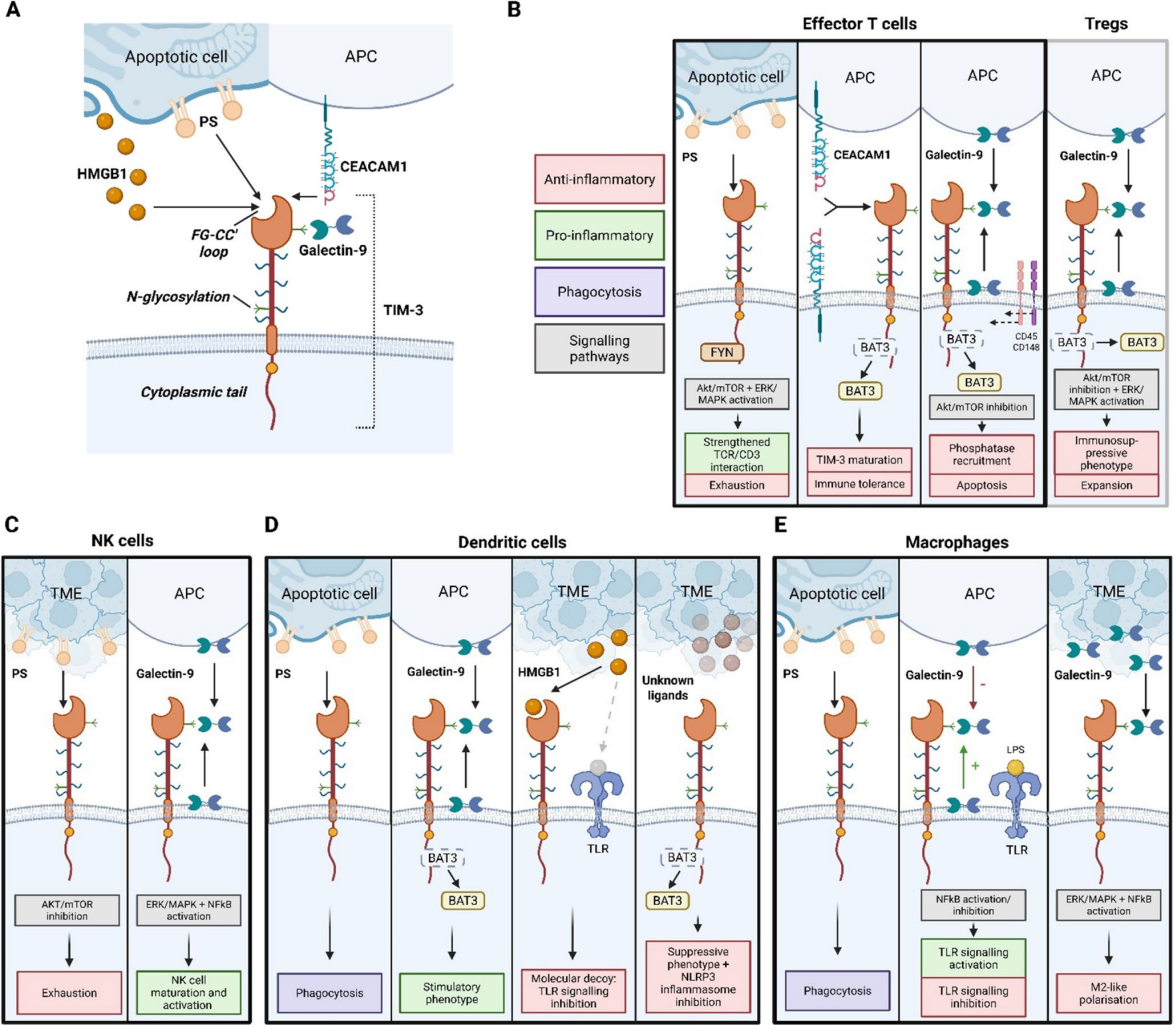
** A** Schematic of TIM-3 and its ligand signalling pathways in immune cells. **B**-**E** Known function and downstream signalling of TIM-3 with its ligands in T cells, NK cells, dendritic cells and macrophages. Abbreviations: APC, antigen presenting cells; LPS, Lipopolysaccharides; NK cells, Natural Killer cells; PS, phosphatidylserine; TME, tumour microenvironment; TLR, Toll-like Receptor; Tregs, T regulatory cells. *Figure was created using BioRender. *

Currently, there are four known ligands for TIM-3; galectin-9, carcinoembryonic antigen-related cell adhesion molecule 1 (CEACAM1), phosphatidylserine (PS) and high mobility group protein B1 (HMGB1), each binding distinct regions of the IgV domain (Fig. 1A) [28–31]. Galectin-9 interacts with the N-linked glycans on the IgV domain, whereas CEACAM1 engages the CC’ and FG loops [31]. PS, binds within a pocket found on all TIM family members in a calcium-dependent manner, a mechanism also suggested for HMGB1. Unlike classical immune checkpoints, TIM-3 exerts both inhibitory and stimulatory functions depending on the kind of immune cell, making its biology complex and only partly understood.

### TIM-3 in Immune Cells

#### Adaptive Immune Cells

##### Effector T Cells

Effector T cells are central to adaptive immunity, consisting of CD4^+^ and CD8^+^ T cells. CD4⁺ T cells differentiate into T helper 1 (Th1) and T helper 2 (Th2) subsets that orchestrate immune responses through distinct cytokine profiles [32]. Th1 cells produce interleukin 2 (IL-2) and interferon-γ (IFN-γ) to promote cell-mediated immunity against intracellular pathogens, whereas Th2 cells secrete IL-4, IL-5, IL-10 and IL-13 to support humoral immunity and defence against extracellular parasites. CD8^+^ T cells directly eliminate infected and malignant cells and secrete cytokines such as IFN-γ to support this response [33]. Given that both CD4⁺ and CD8⁺ T cells were the first identified immune cells to express TIM-3, its regulatory mechanisms have therefore been the most described [34].

Located in lipid rafts, TIM-3 gets recruited to the immunological synapse upon T-cell activation to interact with HLA-B-associated transcript 3 (BAT3) and the tyrosine kinase FYN (Fig. 1B) [33]. In the absence of ligand binding, BAT3 is bound to the cytoplasmic tail of TIM-3, recruiting LCK and allowing T-cell activation [35]. When galectin-9 or CEACAM1 binds, Y256 and Y263 are phosphorylated by the tyrosine kinase ITK, causing BAT3 to be released and allowing TIM-3 to exert its inhibitory function on T cells through the PI3K/Akt/mTOR/NF-kB signalling pathway [35, 36].

## Galectin-9

With BAT3 released, the cytoplasmic tail of TIM-3 becomes available for potential FYN binding [37]. Although the precise function of FYN in this context remains unclear, it is hypothesised that its recruitment could contribute to TIM-3-mediated inhibitory signalling. Analogous to other contexts, FYN may phosphorylate PAG, triggering CSK recruitment and subsequent LCK inhibition, which ultimately dampens T cell receptor (TCR) signalling [37, 38]. Importantly, PAG-associated FYN was also shown to increase intracellular calcium flux, a mechanism that has been observed upon galectin-9 binding to initiate calcium-dependent cell death [32]. Indeed, in vitro studies demonstrated that galectin-9 binding to TIM-3 induced a rapid intracellular calcium influx, activating the calcium/calpain/caspase-1 pathway, which drove Th1 cell aggregation and a combination of apoptotic and necrotic cell death. This mechanism helped to limit Th1 expansion, actively preventing pathological immune activation. The galectin-9/TIM-3 pathway was therefore shown to inhibit T-cell proliferation, reduce cytokine production, and promote T-cell death [39]. Beyond inducing cell death, galectin-9 also triggered another inhibitory mechanism by recruiting the receptor phosphatases CD45 and CD148 into CD3 signalling rafts [33]. Normally excluded from the immunological synapse, their accumulation in the rafts upon TIM-3 cross-linking inhibited LCK and further dampened TCR signalling (Fig.1B).

## CEACAM1

Another key mediator of TIM-3 inhibitory function is CEACAM1, having been implicated in both TIM-3 maturation and signalling [30]. CEACAM1 has been associated with TIM-3 in *cis* during early biosynthesis, enabling surface expression and stability on T cells, while *trans* binding permitted TIM-3 inhibitory activity in T cells by releasing BAT3 [40]. Furthermore, Huang et al. demonstrated in an antigen-specific tolerance mice model that CEACAM1 was essential for proper T-cell tolerance. In a *CEACAM1*^*−/−*^ mouse model, T-cell TIM-3 expression and regulatory cytokines were reduced, leading to hyper-inflammatory responses that were reversed by restoring CEACAM1. Thus, without CEACAM1, TIM-3 levels were decreased, immune activation became unchecked and anti-tumour immunity was enhanced, demonstrating a role promoting immune tolerance in T cells.

Beyond this interaction, the tolerogenic role of TIM-3 is further supported by extensive in vivo and clinical studies. In several autoimmune disease models, including experimental autoimmune encephalomyelitis, inflammatory bowel disease, and autoimmune diabetes, TIM-3 blockade consistently worsened pathology [34, 41, 42]. Additionally, patients with autoimmune conditions such as multiple sclerosis, ulcerative colitis, and psoriasis, exhibited a significantly reduced TIM-3 expression [43–46]. Conversely, in rheumatoid arthritis, patients showed increased TIM-3 expression in peripheral T cells, which inversely correlated with disease severity, suggesting that TIM-3 upregulation in the circulation represents a compensatory response aimed at restraining excessive inflammation [47]. Notably, successful therapies that alleviated autoimmune pathology often restored TIM-3 expression levels [43, 47]. Together, these findings underscore TIM-3 as a critical inhibitory checkpoint required for maintaining immune tolerance and preventing pathological immune activation.

### Phosphatidyl Serine

While TIM-3 is widely recognised for its inhibitory functions, several studies showed that it could also promote T-cell activation under specific conditions. Under acute inflammation, binding of TIM-3 to its ligand PS enhances early TCR/CD3 and CD28 signalling, boosting IL-2 production and activating NFAT, AP-1, and NF-kB in both Jurkat T cells and primary CD4⁺ T cells [37, 48]. Mechanistically, PS binding may allow FYN to phosphorylate TIM-3, promoting recruitment of SH2-containing adaptors such as the PI3K p85 subunit. In this context, PS binding triggers FYN-mediated stimulatory signalling, whereas galectin-9 engagement activates FYN-mediated inhibitory signalling. This phosphorylation-dependent complex activates the PI3K/Akt/mTOR pathway, which in turn amplifies downstream signalling through NFAT, AP-1, and NF-kB, supporting cytokine gene transcription, T-cell survival, and effector differentiation [37]. Consistent with these findings, TIM-3 expression correlated with TCR signalling strength during acute infection, enhancing the Akt/mTOR and ERK/MAPK pathways through S6 phosphorylation [49, 50]. Additionally, in a *listeria monocytogenes* model, TIM-3 was transiently expressed on activated CD8⁺ T cells, and its absence reduced effector responses, including IFN-γ production, highlighting TIM-3 in promoting CD8 + T-cell responses to inflammation [51]. Finally, its dual role is further supported by the TIM-3 signalosome, which contains both inhibitory signalling proteins (CBLB, SHP1, UBASH3A) and stimulatory components (VAV1, LCK, P85A) [52]. Collectively, these findings underscore that TIM-3 can switch from an inhibitory checkpoint to a stimulatory receptor under acute infection, amplifying T-cell activation and effector responses. While TIM-3 can enhance TCR activation during acute infection, excessive activation of the PI3K/Akt/mTOR pathway under chronic inflammatory conditions, such as HIV, hepatitis C virus, lymphocytic choriomeningitis virus or Friend virus, drives terminal differentiation, impairs memory T-cell formation and contributes to T-cell exhaustion [53–57].

Taken together, TIM-3 functions as a context-dependent regulator of effector T cells. Upon galectin-9 or CEACAM1 binding, TIM-3 mediates immune tolerance and limits pathological immune activation through calcium-dependent cell death and phosphatase recruitment. In contrast, PS engagement promotes T-cell activation during acute inflammation, although chronic activation can lead to T-cell exhaustion.

## T Regulatory Cells

T regulatory cells (Tregs) maintain immune balance by suppressing self-reactive lymphocytes to prevent autoimmunity and by restraining effector T cells to limit excessive immune responses [58]. Under normal circumstances, TIM-3 was found to be expressed on only 2–5% of circulating Tregs [59]. However, in the TME, this proportion rose dramatically to 40–60% [59, 60]. Interestingly, these TIM-3^+^ Tregs adopted an effector-like phenotype, characterised by enhanced suppressive capacity [58, 61]. This was mediated through multiple mechanisms, including increased IL-10 production, decreased Akt/mTOR signalling, and increased ERK/MAPK signalling, collectively strengthening Tregs immunosuppressive function (Fig. 1B). In vivo, mice transplanted with tumours and enriched for TIM-3^+^ Tregs showed accelerated tumour growth, whereas Treg-specific TIM-3 knockout resulted in slower tumour progression and reduced CD8 + T-cell exhaustion [61]. These findings indicate that TIM-3 expression on Tregs promotes effector differentiation and enhances their immunosuppressive activity, a contributor to tumour immune evasion. Interestingly, this enhanced suppressive capacity may have been linked to galectin-9 binding, which was shown to promote Tregs expansion [62–64]. By inducing apoptosis in effector T cells while supporting Tregs induction, galectin-9 would help shape the Tregs-to-T-effector cell ratio, contributing to immune homeostasis. However, the precise mechanisms by which galectin-9 regulates Tregs differentiation and function remain incompletely understood [64].

## Natural Killer Cells

Natural killer (NK) cells provide protection against viral infections and contribute to tumour immune surveillance [65]. Their activation is tightly balanced by inhibitory and stimulatory receptors, which together determine the strength of the NK-cell response. Once activated, NK cells mediate their effector functions primarily through the release of lytic granules containing perforin and granzymes, and participate in shaping the adaptive immune response through cytokine production [66].

NK cells exhibit the highest TIM-3 expression among all lymphocytes and TIM-3 is associated with NK cell maturation [65]. TIM-3 is expressed on all mature NK cells and can be induced on immature NK cells in vitro by IL-15 or IL-12 and IL-18 stimulation. However, its function on NK cells remains debated. One study demonstrated that high TIM-3 expression was correlated with enhanced cytokine production and cytotoxicity, whereas cross-linking TIM-3 with antibodies suppressed NK cell-mediated cytotoxicity, implying that activating the TIM-3 pathway through ligand binding can inhibit NK activity [65]. In comparison, Gleason et al. found that galectin-9/TIM-3 binding led to an increase in IFN-y production in NK cells, while blocking this interaction reduced cytokine production [66]. Cross-linking TIM-3 with a similar antibody in this context triggered ERK/MAPK activation and degradation of the NF-kB inhibitor IkBα, promoting NK-cell activation. These conflicting results suggest that the effect of TIM-3 on NK-cell activity might be ligand-dependent (Fig. 1C).

In cancer, elevated TIM-3 levels on NK cells are often linked to impaired anti-tumour responses and poorer prognosis across multiple malignancies, including bladder cancer, lung adenocarcinoma, gastric cancer, melanoma, and esophageal cancer [67–71]. This upregulation of TIM-3 was also linked to NK cell exhaustion in cancers such as melanoma, breast cancer, and esophageal cancer [70, 72]. NK exhaustion was characterised by upregulation of inhibitory receptors, downregulation of IL-2 receptors, functional impairment (including reduced cytotoxicity, cytokine production, and proliferation) as well as decreased expression of activating receptors and key transcription factors [70]. Specifically, in bladder cancer, stimulation of NK cells with cytokines IL-2, IL-15, and IL-21 partially enhanced cytotoxicity, but this effect plateaued, unlike in healthy NK cells [73]. Importantly, TIM-3 blockade restored NK cytotoxicity, consistent with findings in melanoma where TIM-3 inhibition reversed NK cell exhaustion. Mechanistically, TIM-3-mediated NK dysfunction in liver cancer occurred through phosphorylation induced by engagement with its ligand PS, which promoted competitive binding of TIM-3 with PI3K p85 (and p110), thereby inhibiting downstream Akt/mTOR signalling [74]. This disruption impaired NK cytotoxicity abilities and reduced their cytokine production.

Interestingly, in acute myeloid leukaemia (AML), NK cells from patients with early-stage AML (M1 + 2) exhibited high TIM-3 expression that correlated with enhanced effector functions and positive prognostic value [75]. However, NK cells seemed to show signs of dysfunction and even exhaustion in patients with more advanced AML (M4 + 5). The TME could therefore influence TIM-3 role on NK cells, promoting an exhaustive phenotype through PS binding in later stages of cancer.

### Innate Immune Cells

#### Dendritic Cells

Dendritic cells (DCs) are key sentinels of the innate immune system, sensing environmental cues to either promote immune responses or induce tolerance [76]. By presenting antigenic peptides on MHC I and II, activated DCs drive T-cell responses, while also contributing to immune homeostasis by clearing apoptotic cells through phagocytosis [77, 78]. By binding to PS on apoptotic cells, TIM-3 enables DCs to efficiently engulf dying cells (Fig. 1D). Blocking TIM-3 with monoclonal antibodies markedly impairs this phagocytosis and consequently reduces the cross-presentation of apoptotic cell-derived antigens both in vitro and in vivo [78].

Beyond phagocytosis, TIM-3 expression on DCs was also shown to inhibit T-cell stimulation through several mechanisms. In the TME, nucleic acids released by dying tumour cells are critical for initiating anti-tumour immune responses once recognized by pattern recognition receptors (PRRs) such as TLRs [79]. HMGB1, an alarmin that can be released by tumour cells or bind DNA from dying cells, normally facilitates this nucleic acid sensing by TLRs. However, TIM-3 expressed on DCs in the TME can bind HMGB1, effectively preventing its interaction with TLRs. This interaction therefore suppresses nucleic acid-driven activation of innate immunity, limiting effective anti-tumour responses. In addition to acting as a molecular sink, TIM-3 drives DCs toward a suppressive, regulatory phenotype [80]. However, this inhibitory effect is lost upon TIM-3 deletion, which instead enhances anti-tumour responses through NLRP3 inflammasome activation. Consistent with these findings, deletion of BAT3 was also found to drive DCs toward a regulatory-like phenotype, whereas high BAT3 expression was associated with stimulatory DCs [76]. Mechanistically, loss of BAT3 disrupted endoplasmic reticulum homeostasis and rewired cellular metabolism to increase steroid hormone production, with glucocorticoids acting in a paracrine manner to inhibit T-cell activity. TIM-3 therefore serves as an inhibitory receptor on DCs, working as a molecular decoy against HMGB1-TLR interaction and promoting a DC suppressive phenotype through NLRP3 inflammasome inhibition and BAT3 downregulation.

Interestingly, TIM-3 function does not seem to be solely inhibitory in DCs. Anderson et al. showed that upon galectin-9/TIM-3 binding, or with an agonist antibody, DCs had an enhanced proinflammatory cytokine production, such as tumour necrosis factor alpha (TNFα), through the activation of the NF-kB pathway [22]. In contrast, TIM-3-deficient DCs showed markedly reduced cytokine secretion. Thus, TIM-3 function in DC can also promote an inflammatory activity, further highlighting the complexity of this receptor.

### Macrophages

Macrophages are highly plastic innate immune cells, which oscillate between a pro-inflammatory phenotype and an anti-inflammatory/pro-fibrotic phenotype to promote immune homeostasis [81]. Similarly to DCs, they also serve as antigen-presenting cells (APC) and participate in removing apoptotic cells by phagocytosis, maintaining tolerance and preventing autoimmunity (Fig. 1E) [78, 82].

### Crosstalk with Toll-Like Receptors

Crosstalk with TLRs is broadly investigated in macrophage function. Here, TIM-3 is positioned as a critical regulator of macrophage activation, maintaining quiescence by modulating TLR signalling [83, 84]. Under basal conditions, macrophages have been found to have a high TIM-3 expression with low cytokine production. However, upon pathogen recognition via TLRs, the NF-kB pathway becomes activated, resulting in the production of proinflammatory cytokines, such as IL-12, IL-6, and IL-10, while expression on macrophages was markedly downregulated [83]. Overexpression of TIM-3 in macrophages demonstrated its suppressive role in TLR-mediated cytokine production, whereas blocking TIM-3 enhanced macrophage activation. Mechanistically, TIM-3 inhibited LPS/TLR-4-induced NF-kB activation through increased PI3K/Akt phosphorylation and A20 activity, establishing crosstalk between TIM-3 and TLR-4 pathways [84]. It was later shown that TIM-3 effect on TLR signalling was modulated by galectin-9 binding. TIM-3 binding to galectin-9 in *trans* generally suppressed TLR activation (increasing IL-23, reducing IL-12 and STAT1 phosphorylation) whereas *cis* binding supported proper TLR signalling and could upregulate galectin-9 expression [85]. This highlights a context-dependent regulatory role for TIM-3 in macrophages, where its influence on TLR-mediated responses is determined by the mode of ligand interaction. TIM-3 therefore maintains immune homeostasis on macrophages, limiting excessive TLR-mediated inflammation.

### Immunosuppressive Functions in the Tumour Microenvironment

While TIM-3 is often found to boost pro-inflammatory settings, its dysregulation in the TME can be co-opted to favour immune suppression. Its expression on macrophages was consistently associated with a pro-fibrotic phenotype in multiple tumour types, supporting tumour-promoting functions such as migration, invasion, and metastasis in cancers such as hepatocellular carcinoma, anaplastic thyroid cancer, osteosarcoma and glioblastoma [81, 86–88]. Blocking TIM-3 in these macrophages was found to partially reverse the phenotype. Specifically, in glioblastoma, tumour cell-derived galectin-9 promoted this polarisation upon TIM-3 binding, and blockade of this interaction in the TME suppressed tumour growth. Mechanistically, galectin-9/TIM-3 interaction on macrophages engaged STAT1 via its cytoplasmic residues Y256 and Y263, inhibiting STAT1 phosphorylation and nuclear translocation [86, 89]. This reduced miR-155 expression, which normally suppressed SOCS1, a key regulator of pro-fibrotic and anti-inflammatory macrophage polarisation. As a result, IL-10 and arginase-1 (ARG-1) levels increased, driving this macrophage phenotype along in the TME. TIM-3 signalling could also enhance STAT6 and NF-kB pathway activity, further reinforcing their presence [89].

The context-dependent nature of TIM-3 is further highlighted by its contrasting effects in different disease settings. In DSS-induced colitis, TIM-3 limited the differentiation of pro-inflammatory macrophages, and its downregulation or blockade enhanced pro-inflammatory responses, exacerbating tissue inflammation [90]. In contrast, in diabetic nephropathy, TIM-3-expressing macrophages drove pathological changes by activating the NF-kB/TNFα pathway, promoting inflammation and tissue injury [91]. Overall, these findings underscore the context-dependent role of TIM-3 in macrophage biology: while it can restrain excessive pro-inflammatory responses in certain disease settings, in the TME, TIM-3 is co-opted to foster immunosuppression, tumour progression, and metastasis.

### Others: Mast Cells and B Cells

Mast cells are first-line immune cells that respond to allergens and pathogens when antigens cross-link IgE bound to their receptor, FcεRI, triggering degranulation and cytokine production [92]. Mast cells express TIM-3 constitutively, and its expression increases upon antigen and IgE cross-linking, triggering an inflammatory response in a dose-dependent manner [92, 93]. By associating with FcεRI, TIM-3 was found to enhance its downstream signalling pathway, whereas knocking it down rendered mast cells less responsive to this receptor’s cross-linking, resulting in decreased degranulation and cytokine production. While TIM-3 functions in mast cells seemed to enhance their inflammatory responses, in the TME, it appeared to promote an immunosuppressive phenotype. In melanomas, mast cells produced abundant galectin-9, which could bind to IgE, and dampened FcεRI signalling, potentially reducing their pro-inflammatory activation [94]. Galectin-9 was also highly produced by tumour-infiltrating mast cells and was thought to interact with TIM-3 to enhance the expression of CD80, which could interact with the inhibitory receptor CTLA-4 on T cells, thereby promoting local immunosuppression. Additionally, tumour-derived TGF-β upregulated TIM-3 expression on mast cells via the ERK/MAPK pathway [94–96]. This combination of high TIM-3 levels and high galectin-9 signalling seems to shift mast cell function to an inhibitory, pro-inflammatory phenotype, driving tumour growth.

Recently, TIM-3 was detected on B cells, like other inhibitory checkpoints such as TIGIT and LAG-3, although its functional role in B cells remains to be explored [97].

### TIM-3 Implications in Cardiovascular Diseases

The function of TIM-3 is highly context-dependent, depending on the environment, ligand interaction and the cell type by which it is expressed. While its role in cancer and infectious diseases has been extensively studied, its critical role in the cardiovascular field has only recently emerged, with its upregulation in several CVDs, sparking interest in its role in disease progression [25, 26]. Here, we summarise the current knowledge and implications of the TIM-3 pathway in CVDs, highlighting both its protective and potential harmful effects (Table 1).

**Table 1:** Summary of studies shedding light on TIM-3 in cardiovascular diseases. Abbreviations: ADHF, Acute Decompensated Heart Failure; HF, Heart Failure; MACE, Major Adverse Cardiovascular Events; PBMCs, Peripheral Blood Mononuclear Cells; HUVECs, Human Umbilical Vein Endothelial Cells; HASMCs, Human Artery Smooth Muscle Cells; CVB3, Coxsackievirus B3; MI, Myocardial Infarction

Author	Disease	Model	Tissue	Study outcome
Human data				
Meng et al. [26]	Atherosclerosis	Patients (*n* = 84)	Serum	Elevated levels of TIM-3 on CD4^+^ T and CD8^+^ T cells predicted HF prognosis and were associated with increased MACE risks.
Wang et al. [25]	Atherosclerosis	Patients (*n* = 52)	Plasma	TIM-3 levels helped differentiate between HF and non-HF cases in left-to-right shunt congenital heart disease.
Qiu et al. [98]	Atherosclerosis	Patients (*n* = 56)	Lesional artery	PD-1/TIM-3 co-expression on CD8^+^ T cells was associated with an anti-atherogenic environment.
Zhang et al. [23]	Atherosclerosis	Patients (*n* = 75)	Serum	Upregulated TIM-3 expression was associated with disease severity.
Reitsema et al. [101]	Atherosclerosis	Patients (*n* = 37)	Plasma	Increased TIM-3 level trend in female patients was associated with low pro-inflammatory cytokine production.
Li et al. [102]	Atherosclerosis	Patients (*n* = 37)	Serum	TIM-3 expression was significantly upregulated in γδ T cells.
Zhang et al. [103]	Atherosclerosis	Patients (*n* = 46)	PBMCs	Statins reduced TIM-3 expression on patient NK cells.
Hou et al. [24]	Atherosclerosis	Patients (*n* = 63)	PBMCs	Reduced peripheral NK cells with elevated TIM-3 levels.
Lian et al. [99]	Atherosclerosis	Patients (*n* = 5) and patient HASMCs (*n* = 5)	Serum and femoral arterial tissue	Increased serum TIM-3 expression in the lower extremities. Upregulated TIM-3 in HASMCs by PDGF-BB reduced cell proliferation, migration and inflammation.
Yousif et al. [109]	MI	Patients (serum from *n* = 357; MI cardiac tissue from *n* = 2)	Serum and cardiac tissue	Serum levels of galectin-9 and HMGB1 were associated with cardiac remodelling 4 months post-MI. HMGB1 was significantly upregulated in cardiac tissue of patients with previous MI.
**In vivo data**				
Foks et al. [100]	Atherosclerosis	*Ldlr*^−/−^ mice with Western-type diet (*n* = 5)	Aortic root and arch	Galectin-9/TIM-3 interaction was anti-atherogenic by promoting Tregs and B cells.
Frisancho-Kiss et al. [106]	Myocarditis	Male BALB/c mice infected by CVB-3	Cardiac tissue	TIM-3 inhibition reduced CD80 expression on mast cells and macrophages, CTLA-4 levels in CD4^+^ T cells and total Tregs numbers.
Frisancho-Kiss et al. [108]	Myocarditis	Male and female BALB/c mice and TLR-4 defective mice infected by CVB3	Cardiac tissue	Increased TLR-4 levels in male hearts and increased TIM-3 levels in female hearts.
Frisancho-Kiss et al. [107]	Myocarditis	Male and gonadectomised BALB/c mice infected by CVB3	Cardiac tissue	Female-like immune profile in gonadectomised mice; increased IL-4^+^CD4^+^ Th2 cells, Foxp3^+^ Tregs and Tim-3^+^CD4^+^ T cells, with reduced cardiac inflammation and increased M2 macrophages.
Yousif et al. [109]	MI	MI mouse model (*n* = 3)	Cardiac tissue	Increased cardiac HMGB1 6 weeks post-MI.
Huang et al. [110]	MI	MI mouse model (*n* = 6)	Cardiac tissue	Supplementation of Galectin-9 improved tissue remodeling post-MI in Galectin-9-deficient mice and increased M2 macrophage levels
**In vitro data**				
Qiu et al. [104]	Atherosclerosis	HUVECs (*n* = 3)	Lesional artery	TIM-3 was upregulated in HUVECs in response to ox-LDL; limiting apoptosis, restoring cell migration, and suppressing pro-inflammatory cytokines.
Pernaa et al. [105]	Myocarditis	Patient PBMCs (*n* = 1)	PBMCs	Recurrent auto-inflammatory myocarditis in patient with a homozygous loss-of-function mutation in *HAVCR2*.
Hou et al. [24]	Atherosclerosis	NK92 cell line (*n* = 4)	NK cells	TIM-3 blockade prevented TNF-α-induced NK cell death.
Yousif et al. [109]	MI	THP1-derived macrophages (*n* = 6)	Macrophages	HMGB1 induced M1 polarisation of THP1-derived macrophages through the NLRP3 pathway and was prevented TIM-3 pre-incubation.
Huang et al. [110]	MI	Co-culture (RAW264.7; *n* = 6)	Murine primary cardiomyocytes and macrophages	Galectin-9/TIM-3 induced M2 polarisation post-MI; inhibiting PI3K/Akt signalling pathway through TIM-3 on macrophages, driving M2 polarisation post-MI.

### Atherosclerosis

Atherosclerosis is a chronic inflammatory disease in which monocytes and T cells infiltrate arterial tissue, with monocytes differentiating into cholesterol-accumulating foam cells that form atherosclerotic plaques in both coronary and peripheral vasculature [98, 99]. Plaques can cause discomfort, such as claudication in the lower extremities, whereas plaque rupture can result in coronary heart disease by triggering thrombus formation, ultimately causing myocardial infarction (MI) or other ischemic events. TIM-3 was suggested to have a protective role in atherosclerosis across multiple cell types.

### Galectin-9

**Fig. 2:**
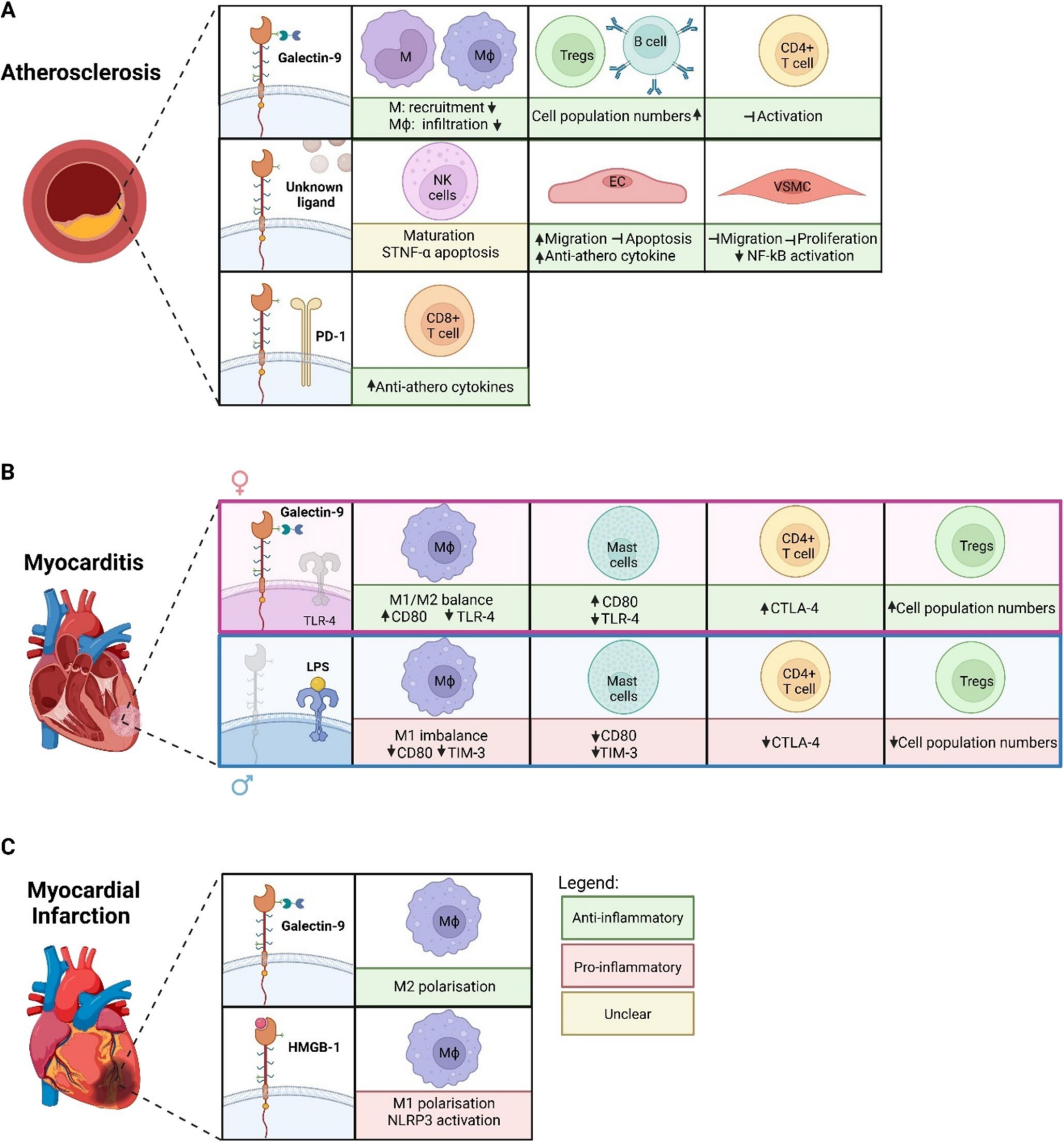
Schematic of known TIM-3 functions in atherosclerosis, myocarditis and myocardial infarction. Abbreviations: M, monocytes; Mϕ, macrophages; Anti-athero; Anti-atherogenic; Tregs, T regulatory cells; NK cells, Natural Killer cells; ECs, Endothelial Cells; VSMCs, Vascular Smooth Muscle Cells. *Figure was created using BioRender.*

In a western-type diet-fed mouse model, anti-TIM-3 antibody treatment increased the plaque formation up to 66% after 8 weeks, specifically in the aorta [100]. Blocking galectin-9/TIM-3 interaction led to reduced Tregs and regulatory B cell populations, which is especially detrimental considering their protective role against atherosclerosis. Indeed, Tregs help maintain an anti-atherogenic environment and stabilise plaques, whereas regulatory B cells secrete IL-10 to suppress pro-inflammatory cytokines and support Treg differentiation, both of which prevent atherosclerosis progression. Moreover, anti-TIM-3 led to increased circulating monocytes and infiltrated macrophages, while also promoting both the number and activity of CD4^+^ T cells. Galectin-9/TIM-3 interaction therefore appears to be a key mechanism limiting atherosclerosis progression, promoting Tregs and B cells, suppressing CD4^+^ T-cell activation, and reducing monocyte recruitment and macrophage infiltration (Fig. 2A). Additionally, this study showed that TIM-3 expression promoted an anti-atherogenic phenotype in B cells, which upon anti-TIM-3 treatment reverted to a pro-atherogenic cell profile.

Consistent with these findings, TIM-3 influence on atherosclerosis progression might be tightly linked to its co-expression with immune checkpoint PD-1 [98]. CD8⁺ T cells co-expressing TIM-3 and PD-1 produce higher levels of anti-atherogenic cytokines than those lacking one or both receptors [98]. Additionally, they have displayed a phenotype resembling that of central memory T cells, known to have a high self-renewal capacity, but also being less cytotoxic. In contrast, simultaneous blockade of both checkpoints resulted in the increased production of both pro-inflammatory cytokine TNFα and IFN-y. Patients with atherosclerosis therefore frequently exhibited CD8⁺ T cells co-expressing TIM-3 and PD-1, suggesting that the combined activity of these checkpoints helps maintain an anti-atherogenic immune environment that slows disease progression (Fig. 2A). Moreover, TIM-3 upregulation on CD4^+^ and CD8^+^ T cells was consistently observed in patients with coronary and peripheral heart disease, supporting the idea that its expression may have limited excessive immune activation and prevented further tissue damage [23, 98, 101–103].

### TIM-3^+^ NK Cells

While TIM-3 expression on effector T cells was associated with an anti-atherogenic environment, its function in NK cells remains ambiguous. Hou et al. reported reduced levels of NK cells in patients with atherosclerosis, but increased TIM-3 expressing cells (Fig. 2A) [24]. TIM-3 expression in turn was associated with risk factors of atherosclerosis as well as circulating inflammatory markers such as TNFα. TIM-3 blockade on NK-cells reduced TNFα–induced cell death of both NK-cell lines and patient-derived NK in vitro, suggesting that TIM-3 may sensitize NK cells to TNFα–induced cell death. While NK-cell death may be beneficial, it is currently debated on whether loss of hyperactive NK cells may limit plaque inflammation. Evidence from Zhang et al., however, suggested that TIM-3 might have been pathogenic in this context [103]. Statin treatment, known for its lipid-lowering and athero-protective effects, reduced TIM-3 expression on patient NK cells. While this could mean that reduced TIM-3 expression of NK cells is beneficial, it may very well be that that TIM-3 only plays a significant role in conditions of high inflammation. In summary, TIM-3 on NK cells appears to regulate their survival and function in a context-dependent manner, with effects that may either restrain or exacerbate atherosclerosis. Further research is needed to clarify how TIM-3 shapes NK-cell behaviour in atherosclerosis.

### Non-Immune Cell Expression of TIM-3

Interestingly, TIM-3 was also expressed on non-immune cells such as endothelial and smooth muscle cells, where it appeared to act as a protective mechanism in the atherosclerotic environment [99, 104]. Stimulation of human umbilical vein endothelial cells (HUVECs) with oxidized low-density lipoprotein (ox-LDL), the key trigger and driver of atherosclerosis, resulted in pro-inflammatory cytokine production and inhibition of cell migration [104]. Additionally, HUVECs upregulated TIM-3 which protected them from ox-LDL-induced apoptosis by activating the JNK pathway and dampening the production of inflammatory cytokine through NF-kB pathway suppression. Specifically, TIM3^+^ HUVECs produced high levels of anti-atherogenic cytokines including IL-10, Il-4 and TGF-β. TIM-3 also reversed the inhibition of migration, vital for maintaining blood vessel wall integrity, promoting repair of damaged endothelial cells and angiogenesis. Blocking TIM-3 expression actively prevented these effects. In this context, TIM-3 appears to protect HUVECs cells against ox-LDL-triggered inflammation, acting as an intrinsic protective mechanism to preserve endothelial function and counteract atherosclerotic damage. Similar to endothelial cells, vascular smooth muscle cells (VSMCs) were shown to express TIM-3 as a protective mechanism. Platelet-derived growth factor-BB (PDGF-BB) promoted atherosclerosis by increasing the production of proinflammatory cytokines in VSMCs resulting in their proliferation and migration to the lesion site [99]. Upon PDGF-BB stimulation, human artery VSMCs (HASMCs) were shown to express TIM-3, which in turn acted as a negative-feedback loop, reducing HASMCs PDGF-BB-induced migration and proliferation. Additionally, overexpressing TIM-3 significantly decreased their proinflammatory cytokine production via the suppression of the NF-kB pathway, consistent with TIM-3 effect on endothelial cells. Thus, TIM-3 may not only affect atherosclerosis progression through the modulation of immune cell function but can also serve as a protective regulator of both endothelial cell and VSMC function (Fig. 2A).

### Myocarditis

Myocarditis is characterised by inflammatory infiltration of the myocardium in response to viral infections, autoimmune activation, or toxic exposures, causing cardiomyocyte damage that may lead to chronic heart failure [105]. Similar to atherosclerosis, TIM-3 appears to play a protective role in myocarditis by regulating the innate immune response. In a CVB3-induced myocarditis model in BALB/c mice, where acute myocarditis developed between days 7–14 post-infection, TIM-3 was expressed on mast cells and macrophages at as early as 6h post-infection, which correlated with increased cytokine production in the heart [106]. Importantly, blocking TIM-3 during the early innate response significantly worsened acute myocarditis at day 12. Mechanistically, anti-TIM-3 treatment reduced CD80 expression on APCs in both the heart and spleen, which in turn decreased CTLA-4 levels on splenic CD4^+^ T cells at 6h post-infection, shifting the balance from immune regulation to immune activation by allowing CD28 co-stimulation. This early disruption of the CD80/CTLA-4 axis during innate immunity subsequently altered the cardiac immune landscape during the adaptive phase, resulting in increased infiltration of CD11b^+^ cells (macrophages and neutrophils) while reducing Tregs populations in the heart. Altogether, these findings indicate that TIM-3 signalling on mast cells and macrophages may protect against inflammatory heart disease by modulating CD80 and CTLA-4 levels during the initial immune response while supporting Treg populations (Fig. 2B). Interestingly, these findings aligned with tumour studies showing that galectin-9 binding to TIM-3 on mast cells enhanced CD80/CTLA-4-mediated immunosuppression [94].

#### Sex-Specific Perspectives of TIM-3

The role of TIM-3 in myocarditis became even more complex when considering sex-specific immune responses, with men exhibiting higher myocarditis incidence and disease severity than women [107]. Frisancho-Kiss et al. investigated whether sex hormones regulate TIM-3 expression using the CVB3-induced myocarditis BALB/c mice model [108]. At day 8 post-infection, females consistently displayed higher TIM-3 expression on mast cells, macrophages and CD4^+^ T cells compared to males. At day 12 post-infection, TIM-3 expression was 122% higher on mast cells from females compared to males, and females exhibited significantly increased CD4^+^TIM-3^+^CTLA-4^+^ T cells and Foxp3^+^ Tregs in the heart. While females exhibited higher TIM-3 expression, males seemed to express higher levels of TLR-4, especially in macrophages. Interestingly, pharmacological blockade of TIM-3 in males exacerbated inflammation and further increased TLR-4 expression while reducing Treg numbers. In contrast, mice with defective TLR-4 signalling showed reduced inflammation accompanied by increased TIM-3 expression on mast cells and macrophages. Together these findings support a cross-regulation between TIM-3 and TLR-4, which depending on their respective expression levels may shift the immune balance towards an anti-inflammatory (high TIM-3, high Tregs) or pro-inflammatory (high TLR-4, low Tregs) environment. In a follow-up study, Frisancho-Kiss et al. further tested the role of sex hormones by performing gonadectomy on male mice [107]. Gonadectomised male mice showed significantly increased TIM-3^+^CD4^+^ T cells, CTLA-4^+^ T cells, and Foxp3^+^ Tregs in the heart, shifting the immune phenotype to resemble that of protected females and reducing myocarditis severity. Testosterone directly suppressed TIM-3 expression on CD4 + T cells, macrophages, and mast cells. Additionally, intact males exhibited predominantly pro-inflammatory macrophages, whereas gonadectomised males showed an even distribution of both pro- and anti-inflammatory macrophages. Taken together, these results demonstrated that by promoting higher TIM-3 expression, which in turn suppressed TLR-4 activity and promoted Tregs, the female hormonal environment supported a more controlled immune response, whereas testosterone-driven TIM-3 suppression, which resulted in TLR-4 upregulation and pro-inflammatory macrophages, predisposed males to more severe myocarditis (Fig. 2B). This TIM-3/TLR-4 cross-regulation could have been directed by galectin-9 binding as demonstrated in later reports in macrophages [84, 85].

The modulatory role of TIM-3 in myocarditis was further illustrated by a 2024 case-report of a young male patient with recurrent auto-inflammatory myocarditis due to a homozygous loss-of-function mutation in *HAVCR2* (c.245 A > G p.Tyr82Cys) [105]. The patient experienced recurrent myocarditis episodes beginning at age four, with complete absence of surface TIM-3 expression on CD4^+^ and CD8^+^ T cells, NK cells, and monocytes. In vitro analysis of PBMCs isolated from the patient revealed excessive TLR4-mediated IL-1β production following LPS/ATP stimulation and unrestrained T-cell proliferation compared to healthy controls, consistent with TIM-3 inhibitory role on TLR-4 activation. In summary, TIM-3 acted as a protective regulator in acute myocarditis by modulating the CD80–CTLA-4 axis, supporting Treg expansion, and suppressing TLR4-driven inflammation, ultimately promoting anti-inflammatory macrophages. Its expression is strongly shaped by the hormonal environment, which shifts the balance between TIM-3- and TLR-4-dominant immune signalling, helping to explain why men experience more severe myocarditis than women.

### Myocardial Infarction

MI occurs when blood flow is obstructed or reduced in the heart, resulting in cell death and scarring which can disrupt cardiac conduction and increase heart failure risk. The role of TIM-3 in MI is highly dependent on the ligand interaction, modulating cardiac inflammation and tissue repair by driving macrophage phenotype switching.

#### HMGB1 Versus Galectin-9

In a recent study, the TIM-3 pathway in human serum, peripheral blood mononuclear cells (PBMCs), and cardiac tissue following MI was investigated [109]. On a transcriptional level, TIM-3 expression was downregulated in T cells within 24h after MI, whereas myeloid cells maintained this expression. In contrast, TIM-3 was significantly upregulated on pro-inflammatory macrophages in the heart, which was predicted to interact with HMGB1 from cardiomyocytes. In vitro analysis revealed that HMGB1 stimulation of THP-1-derived macrophages enhanced NLRP3 inflammasome activation and promoted polarisation toward an *SPP1 +* proinflammatory phenotype and – effects that were prevented by prior TIM-3 blockade [109]. In this context, HMGB1/TIM-3 interaction in macrophages further drives inflammation following MI. Galectin-9, on the other hand, was shown to induce anti-inflammatory effects in macrophages, consistent with previous findings in CVDs [94, 100, 106]. In a murine MI model, galectin-9/TIM-3 binding promoted a more M2-like polarisation, supporting tissue remodelling via the PI3K/Akt pathway, which was reversed upon galectin-9 depletion [110]. In vitro experiments further confirmed that cardiomyocyte-derived galectin-9 drove anti-inflammatory macrophages, suggesting its role in mitigating myocardial remodelling. Taken together, these results indicate TIM-3 possesses opposing roles in MI: promoting inflammation through HMGB1 binding, while also enhancing tissue repair upon galectin-9 interaction (Fig. 2C). Notably, this is supported by observations of Yousif et al. where in a multivariable analysis, serum levels of galectin-9 and HMGB1 at 24 h post-MI were associated with cardiac remodelling 4 months post-MI [109]. Specifically, galectin-9 was associated with a smaller infarct size and HMGB1 with reduced left ventricular ejection fraction. Based on current literature, HMGB1 and galectin-9 do not bind TIM-3 in the same place nor is there any evidence about whether binding of one has a higher affinity or blocks binding of the other. Given their seemingly opposite effects, it would be highly informative to investigate these mechanisms and whether a balance between these two TIM-3 ligands may determine whether MI resolution is successful or progresses to pathological remodelling and heart failure. Furthermore, in the same study it was demonstrated that cardiomyocyte production of HMGB1 was markedly increased in infarcted heart tissue, and actively translocated throughout the cells, where it was predicted to interact with TIM-3 expressed on myeloid cells [109]. Given that HMGB1 is a DAMP released from stressed and dying cells, an increase of HMGB1 from cardiomyocytes is biologically feasible in the MI environment and could represent a marker for acute cardiac damage. Wahid et al. observed significantly increased serum HMGB1 levels in individuals with MI, regardless of whether these patients had heart failure [111]. However, individuals who suffered heart failure without a recent MI did not show the same HMGB1 increase, suggesting that HMGB1 was more closely linked to the acute damage of an MI rather than only long-term damage. Beyond MI, HMGB1 was also reported to be elevated in myocarditis and chronic heart failure, while its inhibition was associated with reduced inflammation and provided protection in various experimental models including sepsis, cardiac and liver ischemia/reperfusion injury, diabetes and autoimmune disease [112]. Taken together, these findings suggest that the TIM-3/HMGB1 axis plays a central role in regulating post-MI inflammation and cardiac remodelling. While HMGB1 is most elevated in MI, its presence in other cardiac and inflammatory conditions suggests that TIM-3 may similarly influence disease processes beyond MI, warranting further investigation.

#### Conclusion and Future Perspectives

TIM-3 has emerged as a multifaceted immune checkpoint. Unlike classical inhibitory receptors, its function is highly context-dependent, capable of exerting both suppressive and stimulatory effects depending on the ligand interaction, the environmental stimuli, and the cell type expressing it.

Beyond its well-established roles in cancer and infectious diseases, TIM-3 functions are similarly complex and disease-specific in the cardiovascular field. In atherosclerosis, TIM-3 predominantly functions as a protective regulator, limiting plaque progression by promoting Treg and B cell populations, restricting CD4 + T-cell activation, and protecting endothelial cells and VSMCs from oxidative stress–induced inflammation. However, its effects on NK cells remain ambiguous, with evidence suggesting both protective and potentially detrimental outcomes, requiring more research. In myocarditis, TIM-3 also serves a predominantly protective role by maintaining the CD80-CTLA-4 axis, supporting Treg populations, and suppressing TLR-4-mediated inflammation. Notably, sex hormones strongly influence TIM-3 expression in myocarditis, with testosterone suppressing TIM-3 while amplifying TLR-4 signalling, thereby predisposing males to increased myocarditis severity. Following myocardial infarction, TIM-3 exhibits ligand-dependent functions: HMGB1 engagement drives pro-inflammatory macrophage and exacerbating cardiac injury, whereas galectin-9 binding promotes anti-inflammatory polarisation and tissue repair. This divergence highlights that ligand availability within the local environment is a key determinant of whether TIM-3 acts in a pathogenic or reparative manner.

Despite these advances, significant gaps remain in our understanding of TIM-3 biology in CVD. First, many studies described TIM-3-mediated effects without identifying which ligand initiated its activation in the given context. Second, although ligand/TIM-3 interactions in effector T cells were relatively well characterised, the downstream signalling pathways and adaptor proteins directly engaged by TIM-3 across different cell types remained poorly defined. Third, TIM-3 function may depend on disease stage or severity. Findings from NK cells in AML and from atherosclerosis studies suggested that TIM-3 could shift from promoting activation to driving exhaustion as disease progressed, which could explain TIM-3 dual role within certain context, highlighting the need for longitudinal and disease-stage-specific investigations. Fourth, the ability of TIM-3 to interact with TLR-4 and FcεRI receptors, further highlights its complex role beyond simple ligand/receptor interaction. It may function as part of a broader regulatory immune network, and investigating its interactions with other immune checkpoints and receptors could clarify some of its unknown mechanistic roles. Furthermore, current studies on TIM-3 focus primarily on atherosclerosis, myocarditis and myocardial infarction. However, evidence from heart failure patients indicated that TIM-3 expression is broadly upregulated across many cardiovascular pathologies, and circulating TIM-3 ligands correlated with worse disease outcomes. Investigating TIM-3 in additional CVD contexts could therefore define its wider relevance and therapeutic potential.

Finally, as anti-TIM-3 therapies advance as next-generation ICI, understanding TIM-3 cardiovascular biology becomes urgent. Given the bidirectional relationship between cancer and CVD – where heart failure patients face elevated cancer risk and cancer predisposes patients to cardiac complications – cancer patients are particularly susceptible to cardiac adverse events, which can be further exacerbated by many cancer therapies [113, 114]. As TIM-3 is ubiquitously expressed, systemic TIM-3 blockade may disrupt cardiac immune homeostasis and predispose patients to cardiovascular immune-related adverse events. These concerns are compounded by significant gaps in our mechanistic understanding, which could result in further unanticipated adverse effects. In recent clinical trials (NCT03489343 and NCT02608268), cardiovascular immune-related adverse events were observed at low rates (e.g. <1% hypertension and < 1% MI within 24 months). However, further investigation in larger cohorts with a more favourable set-up (i.e. an exclusion criteria for NCT03489343 was clinically significant cardiovascular disease or condition, and longer follow-up) is required to fully grasp the implications of TIM-3 ICI. This underscores that understanding TIM-3 mechanisms in the cardiovascular system is critical not only for predicting and mitigating adverse effects in patients undergoing anti-TIM-3 cancer therapy but also for elucidating the pathways that drive CVD development and progression.

## Key References

- Wolf Y, Anderson AC, Kuchroo VK. TIM3 comes of age as an inhibitory receptor. Nature Reviews Immunology 2019 20:3. Nature Publishing Group; 20:173–85.
  - This review summarized, for the first time, contemporary knowledge on TIM-3 including its function in/on immune-cell types other than just T cells.

- Zhang N, Zhang M, Liu R-T, Zhang P, Yang C-L, Yue L-T, et al. Statins reduce the expressions of Tim-3 on NK cells and NKT cells in atherosclerosis. Eur J Pharmacol. 2018;821:49–56.
  - This article not only described a role for TIM-3 in atherosclerosis but also pointed out the effect of guideline-directed medical therapy for cardiovascular disease.

- Pernaa N, Vakkuri A, Arvonen M, Kuismin O, Santaniemi W, Glumoff V, et al. Germline HAVCR2/TIM-3 Checkpoint Inhibitor Receptor Deficiency in Recurrent Autoinflammatory Myocarditis. J Clin Immunol. 2024;44:81.
  - While the immune system is an adapative player, assessing immune checkpoint-related genetic components that could predispose patients to a dysregulated immune system is where we believe the field should be focussing on.

- Yousif LI, Sijtema AG, Appels Y, Screever EM, van Blokland I V., Oelen R, et al. The immune checkpoint TIM-3/HMGB-1 axis in myocardial infarction. npj Cardiovascular Health 2025. Nature Publishing Group; 2:1–12.
  - This manuscripts reveals how TIM-3 interaction with HMGB1, released as a DAMP from injured cardiomyocytes, can induce highly inflammatory macrophages. This positions TIM-3/HMGB1 interaction as a therapeutic target to prevent excessive inflammation.

- Huang J, Hu W, Xiong H, Zhou Y, Cao F, Ding C, et al. Cardiomyocyte-derived Galectin-9 induces macrophage M2 polarization via the TIM3 pathway to attenuate myocardial remodeling post-myocardial infarction. Mol Cell Biochem . 2025;480:4809–27.
  - This manuscript in turn shows the other side of TIM-3 – how galectin-9 binding polarizes macrophages towards more homeostatic macrophages after an infarction. This ties in with the findings of the previous key reference where galectin-9 levels in serum of patients post-myocardial infraction were associated with a smaller infarct size after 4 months.

## Data Availability

No datasets were generated or analysed during the current study.
